# Screening for Depression and Anxiety Using a Nonverbal Working Memory Task in a Sample of Older Brazilians: Observational Study of Preliminary Artificial Intelligence Model Transferability

**DOI:** 10.2196/55856

**Published:** 2024-12-12

**Authors:** Alexandra Livia Georgescu, Nicholas Cummins, Emilia Molimpakis, Eduardo Giacomazzi, Joana Rodrigues Marczyk, Stefano Goria

**Affiliations:** 1 thymia, International House, 64 Nile Street, London, N1 7SR, United Kingdom, 44 7477285252; 2 Department of Psychology, Institute of Psychiatry, Psychology & Neuroscience, King’s College London, London, United Kingdom; 3 Department of Biostatistics and Health Informatics, Institute of Psychiatry, Psychology & Neuroscience, King's College London, London, United Kingdom; 4 CAMHS Digital Lab - Department of Child and Adolescent Psychiatry, Institute of Psychiatry, Psychology & Neuroscience, King's College London, London, United Kingdom; 5 Grupo Laços Saúde, Rio de Janeiro, Brazil

**Keywords:** depression, anxiety, Brazil, machine learning, n-back, working memory, artificial intelligence, gerontology, older adults, mental health, AI, transferability, detection, screening, questionnaire, longitudinal study

## Abstract

**Background:**

Anxiety and depression represent prevalent yet frequently undetected mental health concerns within the older population. The challenge of identifying these conditions presents an opportunity for artificial intelligence (AI)–driven, remotely available, tools capable of screening and monitoring mental health. A critical criterion for such tools is their cultural adaptability to ensure effectiveness across diverse populations.

**Objective:**

This study aims to illustrate the preliminary transferability of two established AI models designed to detect high depression and anxiety symptom scores. The models were initially trained on data from a nonverbal working memory game (1- and 2-back tasks) in a dataset by thymia, a company that develops AI solutions for mental health and well-being assessments, encompassing over 6000 participants from the United Kingdom, United States, Mexico, Spain, and Indonesia. We seek to validate the models’ performance by applying it to a new dataset comprising older Brazilian adults, thereby exploring its transferability and generalizability across different demographics and cultures.

**Methods:**

A total of 69 Brazilian participants aged 51-92 years old were recruited with the help of Laços Saúde, a company specializing in nurse-led, holistic home care. Participants received a link to the thymia dashboard every Monday and Thursday for 6 months. The dashboard had a set of activities assigned to them that would take 10-15 minutes to complete, which included a 5-minute game with two levels of the n-back tasks. Two Random Forest models trained on thymia data to classify depression and anxiety based on thresholds defined by scores of the Patient Health Questionnaire (8 items) (PHQ-8) ≥10 and those of the Generalized Anxiety Disorder Assessment (7 items) (GAD-7) ≥10, respectively, were subsequently tested on the Laços Saúde patient cohort.

**Results:**

The depression classification model exhibited robust performance, achieving an area under the receiver operating characteristic curve (AUC) of 0.78, a specificity of 0.69, and a sensitivity of 0.72. The anxiety classification model showed an initial AUC of 0.63, with a specificity of 0.58 and a sensitivity of 0.64. This performance surpassed a benchmark model using only age and gender, which had AUCs of 0.47 for PHQ-8 and 0.53 for GAD-7. After recomputing the AUC scores on a cross-sectional subset of the data (the first n-back game session), we found AUCs of 0.79 for PHQ-8 and 0.76 for GAD-7.

**Conclusions:**

This study successfully demonstrates the preliminary transferability of two AI models trained on a nonverbal working memory task, one for depression and the other for anxiety classification, to a novel sample of older Brazilian adults. Future research could seek to replicate these findings in larger samples and other cultural contexts.

## Introduction

### The Problem of Underdiagnosing Depression and Anxiety in the Older Population

Major depressive disorder (MDD) and generalized anxiety disorder (GAD) are two of the most common mental health conditions and are leading causes of disability and premature mortality. Depression is ranked by the World Health Organization as the single largest contributor to global disability (accounting for 7.5% of all years lived with disability in 2015), while anxiety ranks sixth (3.4%) [1].

Depression and anxiety present intricate challenges in diagnosing and treating older individuals. With depression ranking among the most prevalent psychiatric disorders in this demographic [2], its impact on disability and mortality cannot be understated [3]. Importantly, approximately half of the cases remain undiagnosed, unveiling a critical gap in recognition and intervention [2,4]. Notably, an established and recurring observation is the strong association between late-life MDD and GAD, emphasizing the comorbidity and overlap between these two conditions [5,6].

Barriers to diagnosing depression and anxiety in the older population can be summarized as a combination of patient-related and clinician-related factors. On the patient side, people may not be checking in with their clinicians when necessary, due to insufficient health literacy [7], the stigma surrounding mental illness, or even the focus on other chronic physical health issues, which impedes self-reporting of mental illness symptoms [8]. Perhaps, even before presenting for a checkup, various beliefs might lead people to think that their symptoms do not warrant attention, or they may fear medication or may lack belief in the efficacy of the available treatments or interventions [9]. On the clinician side, primary care clinicians who are responsible for recognizing and treating mental illnesses in patients often do not have the necessary training to identify symptoms in the older population because they overlap with and are masked by normal age-related changes or somatic expressions [10]. A meta-analysis showed that primary care providers detected only 40% to 50% of depression cases and that they were less successful in detecting depression among older adults than among younger adults [4]. Moreover, clinicians are overworked and often do not have the bandwidth to check in regularly or to ask about mental health during physical or routine checkups, if the topic of mental health is not addressed by the patient themselves [11].

In addition to timely detection of mental illness symptoms, tracking mental health over time in the older population is important for two key reasons. First, depression and anxiety in older adults are often chronic or recurrent [6]. Second, it is imperative to track treatment efficacy; older individuals with depression frequently experience heightened drug sensitivity due to age-related changes in drug processing and multiple medication use [12]. Consequently, adjustments in antidepressant drug utilization are necessary to prevent adverse reactions and side effects.

Taken together, the abovementioned complexities contribute to the pervasive underreporting and mismanagement of depression and anxiety among the older population. Given the older population and the growing importance of mental health in older individuals, there is a compelling need for accurate early diagnoses and better remote monitoring of depressive and anxiety symptoms in these patients. Such monitoring may plausibly enhance the overall well-being of the older population (but this would need to be demonstrated in clinical trials). The use of remote measurement technologies (RMTs) in monitoring depression and anxiety could offer considerable benefits. RMT enables continuous, real-time symptom tracking by collecting various types of data, including even those from wearable devices and smartphone technology. This approach, as shown in the Remote Assessment of Disease and Relapse in Major Depressive Disorder (RADAR-MDD) study, is characterized by high adherence, making it a practical, accessible, and engaging method for patients [13]. However, adherence in the general population (without a diagnosis of major depression) still needs to be investigated.

### The Promise of Using Artificial Intelligence for Remote Mental Health Screening

The utilization of artificial intelligence (AI) in remote screenings for depression is a promising avenue in mental health care [14]. AI algorithms can analyze vast datasets of patient information (collected in person or using RMTs), including self-reported symptoms, physiological markers, and even speech or text patterns, to assist in the early detection of MDD and GAD and the tracking of their symptoms. Speech analysis, in particular, has been a prominent area of investigation, examining patterns in tone, cadence, word choice, and linguistic cues that could signal underlying psychological distress or disorders [15]. Additional dimensions such as keystroke patterns, social media interactions, and even eye or body movement patterns captured through wearable devices are emerging as potential behavioral biomarkers [16]. These biomarkers can then be used as input features for machine learning models. However, the pursuit of comprehensive and reliable behavioral biomarkers remains ongoing because most biomarker investigations are not ready for use in clinical practice or are not generalizable [16]. Moreover, most existing voice-based depression datasets comprise speakers younger than 60 years [17].

The n-back task is an experimental cognitive assessment often used in psychological research to evaluate working memory and cognitive control [18]. Participants are required to continuously monitor and compare a series of stimuli, typically numbers, letters, or shapes, and indicate when the current stimulus matches the one shown “n” steps earlier in the sequence, making it a useful task in a cross-cultural, nonverbal context. While not a direct diagnostic tool, performance in the n-back task offers insights into working memory dysfunction in depression and anxiety. For example, a recent systematic review concluded that 2-back task performance is especially significantly different in depression cases compared to controls [19]. The n-back task has been used by the authors before in training an AI model to detect depression and its symptoms [20,21]. It showed, among other things, that somatic and psychomotor symptoms are more strongly associated with n-back performance scores.

### The Goal of This Study

This paper aims to assess the performance of two binary classification AI models developed by thymia [22], a company that develops AI tools for mental health and well-being assessments, in order to screen for depression and anxiety. The models were initially trained on n-back performance features collected remotely from a large diverse population (from mainly the United Kingdom and the United States, and a smaller proportion from Spain, Mexico, and Indonesia, aged 18 to 80 years). In this study, we evaluated them in a completely new dataset, which comprises an older Brazilian population, recruited with the help of Laços Saúde, a company specializing in nurse-led, holistic home care. It is based on the Dutch Buurtzorg model [23], which supports independent living in a community setting.

## Methods

### Recruitment

This analysis is part of a larger longitudinal research study. Participant inclusion criteria for the larger study were: (1) being older than 18 years of age; (2) receiving ongoing health support via Laços Saúde; (3) having normal or corrected-to-normal vision; (4) having normal hearing; (5) being fluent speakers of Brazilian Portuguese; (6) being able to read, understand, and sign the instructions and informed consent form; (7) having access to a laptop, smartphone, tablet, or other device and being able to use them; and (8) being willing to be audio and video recorded (because the data collection involved the collection of speech samples and facial expressions). For the current analysis, we included only participants above the age of 50 years who had completed all activities (ie, the n-back tasks and the administered questionnaires) at least once (since the larger study was arranged as a longitudinal study).

### Ethical Considerations

The ethical review process for this study was led by an independent research ethics expert (Dr. David Carpenter) with an approval on August 11, 2022, on behalf of the Association of Research Managers and Administrators. Participation was voluntary and based on informed consent, and participants were not offered any compensation, in line with the Brazilian Federal Constitution of 1988. All participants read an information sheet and had the opportunity to ask questions before signing an online consent form. The information sheet assured participants that their data would be stored safely and securely, pseudonymized, and processed under the terms of UK data protection law (including the UK General Data Protection Regulation and the Data Protection Act 2018). After informed consent was obtained digitally (via a downloadable copy), participants could proceed to the thymia dashboard with the activities.

### Laços Saúde Protocol

The study was retrospectively (after data collection started) registered at the International Standard Randomized Controlled Trial Number Registry (registration number: ISRCTN90727704) due to the fact that it represented a clinical study and a validation of an existing AI model in a new sample.

Participants were sent an invitation via WhatsApp from Laços Saúde, with a link to the study description and invitation from thymia. Participants had the chance to ask questions to the thymia researchers via email and could then give online consent for participation. After consenting, participants proceeded to the thymia dashboard, which had a demographics questionnaire assigned to them. After completing it, participants received a link to the thymia dashboard every Monday and Thursday for 6 months via WhatsApp. The dashboard had a set of activities assigned to them that took 10-15 minutes to complete. Activities were presented in the same order on the dashboard but there was no instruction as to the required order of completion, and it cannot be excluded that participants may have completed the activities in a different order.

thymia’s n-back implementation is a card memory game lasting 5 minutes, where a card, with a rounded edge and a centrally presented digit (from 1-5), appears briefly on the screen, alternated with empty intervals. Participants tap or click (depending on the device used) when the current character matches the one from “n” cards ago. We implemented two memory load versions as levels with two shorter practice blocks: a 1-back and a 2-back level. Each had two main blocks (ie, sequences of 13 and 14 card stimuli, respectively). Of these, 25% were targets, generated pseudorandomly with certain rules: (1) ensuring a roughly even distribution of all match numbers was present across blocks, (2) ensuring match positions were roughly equally distributed across all blocks, and (3) ensuring that two consecutive blocks did not have a target on the same match position. The stimulus presentation duration was 400 milliseconds, and the time until the next card was presented was 900 milliseconds. Participants were instructed to tap or click on the card when it matched the one before or two before, respectively. The participant could start responding as soon as the letter was presented; they could also respond for 500 milliseconds after it was presented. Feedback was shown as “correct” or “incorrect” for false alarms (commission errors). thymia’s platform is able to collect n-back metrics on laptops or mobile devices with 10 millisecond precision for reaction times, in line with the best performers of online studies platforms [24].

The Patient Health Questionnaire (8 items) (PHQ-8) is a shortened version of the PHQ-9 [25] that excludes the question on suicidality and self-harm, which cannot be ethically asked in an unsupervised, remote online context. It uses a 10-point cutoff for depression and has been validated in a geriatric sample in a primary care setting [26].

The Generalized Anxiety Disorder Assessment (7 items) (GAD-7) is a questionnaire designed to evaluate the frequency and severity of anxiety symptoms experienced over the past 2 weeks [27]. It uses a 10-point cutoff for anxiety. It has been previously validated in a geriatric sample [28].

The relevant schedule for this analysis was as follows: among the weekly activities assigned for Monday were the n-back memory card game activity and questionnaires on the patient’s current state (eg, tiredness, mood, contextual variables, possible confounders such as coffee consumption) and medication adherence. Every 2 weeks on Monday, the GAD-7 and PHQ-8 were added to the assigned activities on the dashboard.

### N-Back AI Models

The thymia proprietary dataset is a longitudinal dataset with over 6000 participants from mainly the United Kingdom and the United States, and a smaller proportion from Spain, Mexico, and Indonesia, aged 18 to 80 years with a mean of 35 years.

While completing a session of the thymia n-back game, timestamps of all clicks and taps on screen (depending on the device used) were recorded; the clicks were matched against the correct response (no click if the card was not a match or a click otherwise) and a number of metrics were calculated. In particular, 3 sets of metrics were produced: (1) an overall set that considered the entire session, (2) a set that only considered the 1-back part of the experiment, and (3) a set that only considered the 2-back part of the experiment. In particular, for every set, the average reaction time was calculated as the time from when a card was presented to when a click occurred. The false-positive rate was the average number of errors, and similarly, precision and recall were computed by looking at the average number of correct versus incorrect clicks. We will refer to these sets of features as n-back biomarkers.

thymia’s predictive models for depression and anxiety use, on top of the n-back biomarkers, other features to account for confounders: age, gender, device type, and game exposure frequency. The modeling task was presented as a binary classification task using PHQ-8 scores ≥10 and GAD-7 scores ≥10 as thresholds for the target label. The models themselves were Random Forest models [29] with 100 trees and a maximum depth of 3; there was no model search or meta-parameter optimization since the aim of this study was to investigate whether the signal transfers from one population to the target one without introducing model or meta-parameter complexities.

### Hypotheses

We expected that there would be a significant difference in terms of key n-back parameters (lower average precision, higher average false-positive rate, and longer average reaction time) for 2-back compared to 1-back tasks in the older Brazilian sample, which would be comparable to a previous control sample of n-back data (Hypothesis 1).

We also hypothesised that the thymia AI models’ evaluation in the older Brazilian sample would achieve a value of at least 0.7 area under the receiver operating characteristic curve (AUC; ie, a common textbook metric as a graphical representation that illustrates the performance of a binary classification model considered as “acceptable discrimination” [30], generally accepted in clinical AI research [31]) and 65% sensitivity (also known as the true-positive rate, measuring the proportion of actual depressed or anxious cases correctly identified) and specificity (true negative rate, measuring the proportion of actual nondepressed or nonanxious cases correctly identified) (Hypothesis 2).

## Results

### Demographics

**Figure 1.**
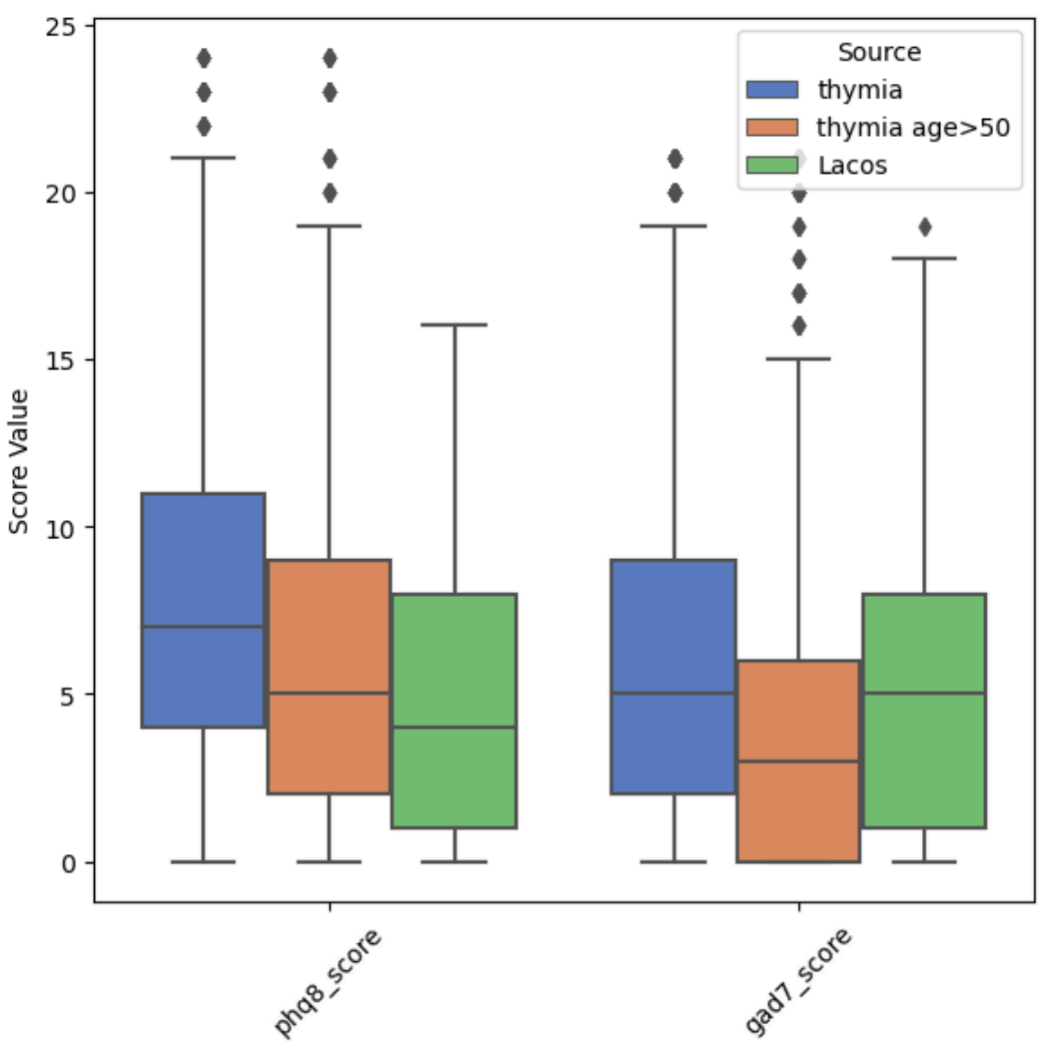
PHQ-8 and GAD-7 score distributions (median value as the horizontal line, interquartile range, minimum and maximum data values as whiskers, and outliers as rhombus shapes) in the full thymia dataset (blue), thymia’s >50 years of age subset (orange), and Laços Saúde patients (green). GAD-7: Generalized Anxiety Disorder Assessment (7 items); PHQ-8: Patient Health Questionnaire (8 items).

The subset of the data used in this report from the larger Laços Saúde longitudinal study on the thymia platform consisted of 69 patients aged 51 to 92 years, with a mean age of 71 years. Participants were included in this sample if they had at least 1 completed instance of the n-back task (1-back and 2-back) and GAD-7 and PHQ-8 questionnaires (within the same week). The distributions of depression and anxiety target scales can be found in Figure 1.

### N-Back Data: Descriptive Analysis

**Figure 2.**
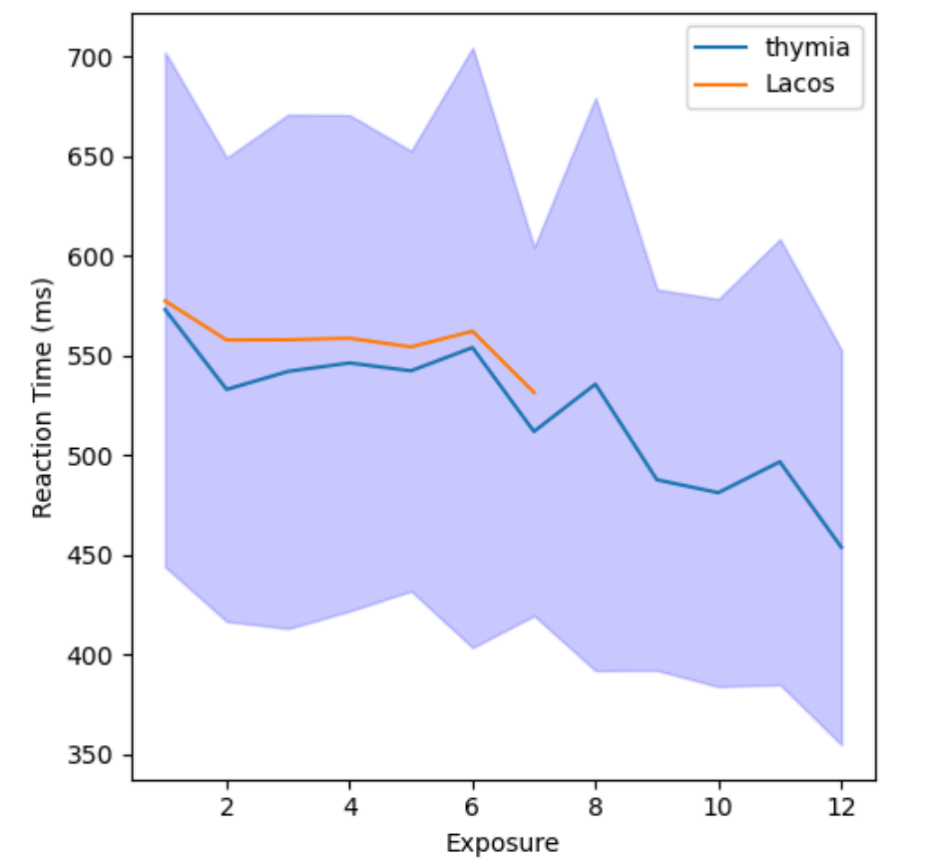
Visualization of the 2-back game learning rate curve: the graph shows a comparison of average reaction time in milliseconds (on the y-axis) for the 2-back game relative to the number of exposures a participant had longitudinally (on the x-axis). The data contrast thymia’s subset of participants aged over 50 years (blue line) with the Laços Saúde sample (orange line). The shading represents CIs as error bars. Notably, while the Laços Saúde curve is shorter due to fewer participants completing the task multiple times, it remains comparable to the thymia curve, which is truncated at 12 iterations for clarity.

**Figure 3.**
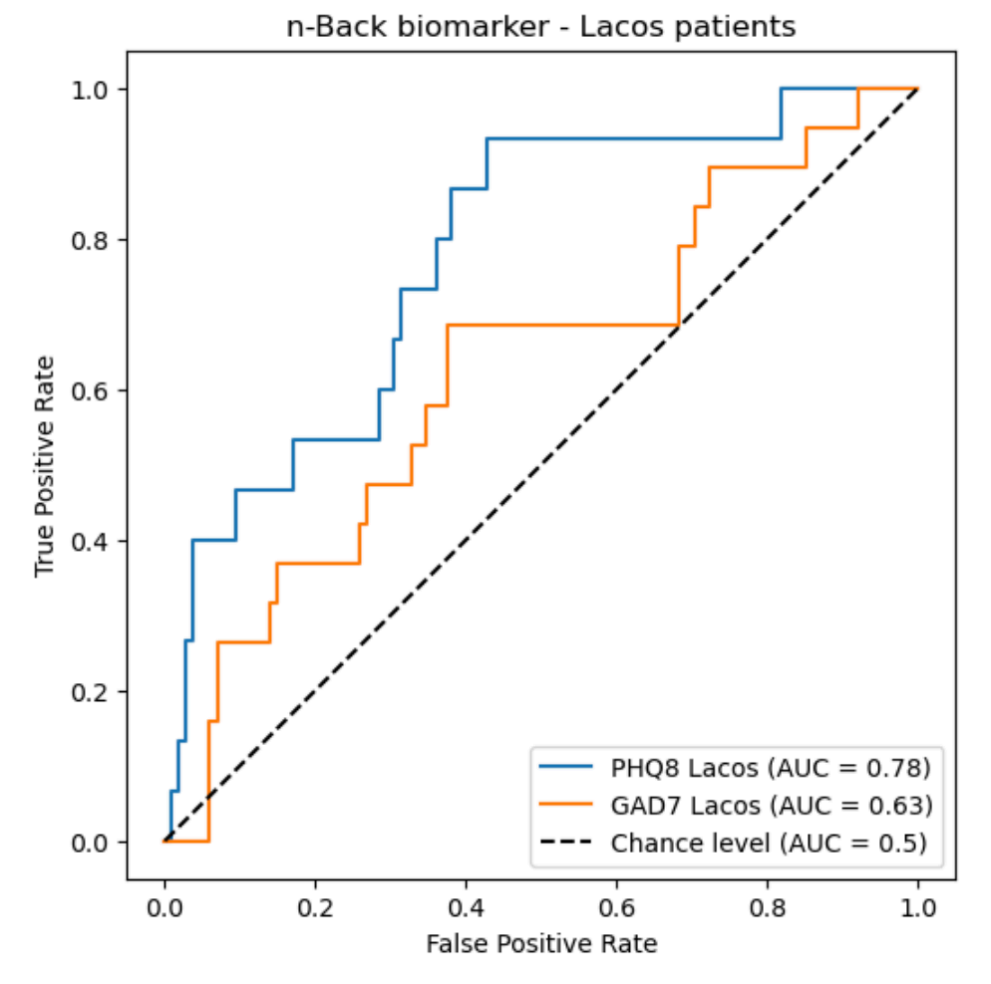
Receiving operating curve with corresponding AUC of the scores from the PHQ-8, for depressive symptoms, and the GAD-7 binary predictive models using the memory card game n-back performance features (reaction time, false-positive rate, and precision and recall) as input. AUC: area under the receiver operating characteristic curve; GAD-7: Generalized Anxiety Disorder Assessment (7 items); PHQ-8: Patient Health Questionnaire (8 items).

Before utilizing thymia’s models on the new dataset, we conducted a series of tests to evaluate the quality of the study’s collected data. Specifically, we aimed to determine the deviation of certain key n-back metrics from our benchmark thymia dataset. We observed that: (1) the stimuli sequences frequency was in line with our expectation of 2 seconds; (2) the learning effect was in line with our expectations (Figure 2); (3) average reaction times for 2-back tasks were significantly higher than 1-back tasks; (4) the average false-positive rate for 2-back tasks was roughly double than that for 1-back tasks; and (5) the average precision for 2-back tasks was significantly lower than 1-back tasks.

From Table 1, we also noticed that the average task performance dropped as expected for an older population, suggesting that models trained on a younger average population might be able to extrapolate to the older one. A mixed ANOVA was run using the Pingouin statistical package in Python 3 [32] to test Hypothesis 1, which found that, indeed, there was a significant difference in the expected direction between 2-back and 1-back tasks for reaction times (*F* [1,2196]=1748.05, *P*<.001, η_p_^2^=0.44), false-positive rates (*F* [12,206]=1407.3, *P*<.001, η_p_^2^=0.39), and precision rates (*F* [12,196]=1980.15, *P*<.001, η_p_^2^=0.47).

**Table 1.** Average RTs^a^ in milliseconds, FPRs^b^, and P^c^ rates for the 2B^d^ and 1B^e^ memory card game for the 3 datasets: thymia, the thymia subset of participants who were over the age of 50 years, and the Laços Saúde patients.

Metrics	thymia dataset	thymia’s >50 years of age dataset	Laços Saúde dataset
RT-2B, ms	484	531	576
RT-1B, ms	425	471	557
FPR-2B	0.09	0.1	0.13
FPR-1B	0.06	0.03	0.06
P-2B	0.77	0.75	0.59
P-1B	0.93	0.92	0.75

a RT: reaction time.

b FPR: false-positive rate,

c P: precision.

d 2B: 2-back.

e 1B: 1-back.

### N-Back Biomarkers’ Performance on Laços Saúde Data

To evaluate the transferability of predictive power from two models (depression and anxiety screening) developed using the thymia internal dataset using n-back features to the older Brazilian population, we tested on the Laços Saúde patient cohort. Specifically, we chose models designed to classify depression and anxiety based on thresholds defined by PHQ-8 scores ≥10 and GAD-7 scores ≥10, respectively. The test results, depicted in terms of the AUC, can be found in Figure 3.

**Figure 4.**
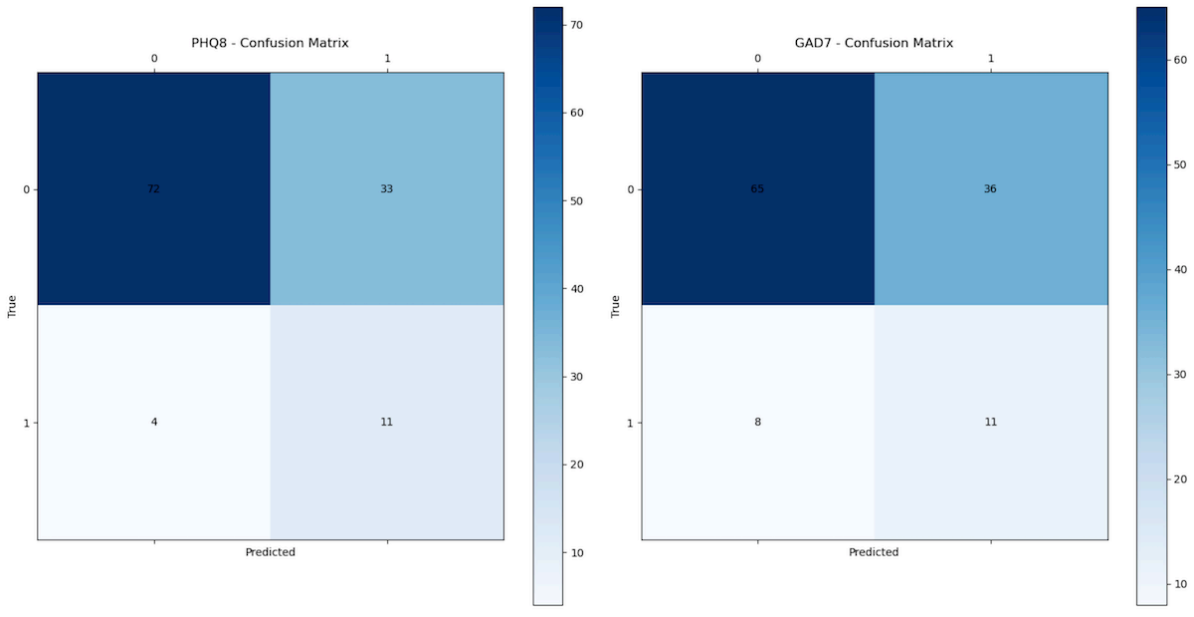
Confusion matrices for the PHQ-8 (left) and GAD-7 (right) score classification. On the “Predicted” axes of “True” and “Predicted” (cases), 0 is subthreshold and 1 is above-threshold. GAD-7: Generalized Anxiety Disorder Assessment (7 items); PHQ-8: Patient Health Questionnaire (8 items).

The depression classifier yielded an AUC of 0.78; when we selected a probability threshold to achieve the most balanced results for the true-positive rate and true negative rate, we got a specificity reaching of 0.69 and a sensitivity of 0.72. The anxiety classifier yielded an AUC of 0.63; when we selected a probability threshold to achieve the most balanced results for the true-positive rate and true negative rate, we got a specificity reaching of 0.58 and a sensitivity of 0.64. In Figure 4, we see the confusion matrices for the selected probability threshold.

All of the above metrics summarize the performance of the models at an aggregate level and every data point is considered as an independent observation. However, since the study was longitudinal, we did have participants who performed the test multiple times, so observations from the same participant were not really independent. In order to make sure that the result would hold when enforcing independence of the observations, we also reran the AUC scores on a subset of the data that only had results from the first time participants were exposed to the n-back task. Here we found AUCs of 0.79 for PHQ-8 and 0.76 for GAD-7, confirming that the quality of the signal was not an artefact of the unrealized independence assumption.

## Discussion

### General Findings

This study demonstrates the preliminary transferability of the performance of two AI models (1 for binary depression classification and 1 for binary anxiety classification) to a new dataset of older Brazilian adults, vastly distinct from the proprietary thymia data the models were initially trained on. To our knowledge, this is the first time such transferability of an AI model for detecting depression using features from a cognitive game across different datasets has been reported. The performance of a depression detection model is dependent on various factors like the dataset size, preprocessing strategy, feature engineering, and the model itself, which are all determined by the dataset used for training, often not generalizing well to other datasets [33].

The depression classification model demonstrated robust performance with an AUC of 0.780 (95% CI 0.650, 0.871), based on 100 bootstrapped samples, a specificity of 0.69, and a sensitivity of 0.72. This performance is significantly above chance level, as evidenced by the lower bound of the CI exceeding 0.5, which represents chance performance, and aligns with our primary hypotheses. Notably, values closer to 1.0 are ideal for sensitivity and specificity, minimizing false negatives and positives, respectively. An AUC above 0.5 suggests better-than-random discrimination, with our model’s AUC falling in the acceptable range of 0.70 to 0.80 [30]. The above-chance AUC suggests that the model is capable of distinguishing between the target and nontarget classes at a level better than random guessing.

In contrast, the anxiety classification model had an AUC of 0.63, a specificity of 0.58, and a sensitivity of 0.64. However, when assessing independence in the longitudinal dataset and analyzing first-time n-back task exposures, the AUC scores improved to 0.76. This suggests that for anxiety scores, while the cross-sectional results were promising, the model underperformed longitudinally with the smaller dataset.

As a benchmark model, we also tested a model that used only age and gender as the input, and we got AUCs of 0.47 for PHQ-8 and 0.53 for GAD-7. This suggests that our model has indeed learned the relationship between n-back tasks and PHQ-8 and GAD-7.

Potential reasons for the two models’ transferability to the new demographic are related to the use of n-back features, considering that the n-back task is a widely used nonverbal working memory task used across cultures. Importantly, the n-back task has been used before in an AI model to detect depression and its symptoms [20], where it showed that somatic and psychomotor symptoms are more strongly associated with n-back performance scores. This is relevant because older individuals tend to exhibit more somatic symptoms compared to younger populations [10], hence n-back biomarkers may be able to capture that.

The two models are expected to perform even better when they are fine-tuned with data representing the new demographics (ie, including them in the training set) and when additional behavioral biomarkers are added. Among these, 1 biomarker that has been receiving a lot of attention is speech [15,20,21]. However, for a model to be generalizable to other cultures and languages, the speech biomarkers need to be culturally invariant. An additional problem is that there are known speech differences between younger and older speakers [17,34], which, again, will have implications for the retraining of the models.

### Implications

There are several benefits in deploying culture-agnostic AI models in real-world clinical settings to detect mental health issues among older adults. A recent analysis in Brazil uncovered a depression underdiagnosis prevalence of 63.6% [35]. One of the groups in which they found a high discrepancy is among older participants: people older than 59 years are less likely to receive a depression diagnosis in comparison with all other age categories even after controlling for confounders. They also report higher prevalence for underdiagnoses in participants reporting only 1 medical appointment in the last 12 months. They identify that a percentage of underdiagnosed depression cases in Brazil in 2019 could have been prevented by access to health visits (5.59%) and addressing barriers for depression diagnoses among older individuals (3.32%) [35]. Therefore, tools that allow for remote screening and remote monitoring could potentially aid in reducing the underdiagnosis of depression (and anxiety) in Brazil and beyond. Given the engaging nature of gamified cognitive tasks like the n-back memory game, and given that it takes only 5 minutes to complete (not significantly longer than completing 2 standard clinical questionnaires), we believe that an AI tool based on the n-back task has the potential for widespread use, beyond users who may already be playing cognitive games. Future studies will need to further validate the efficacy, feasibility, and accessibility of this approach in broader real-world settings in randomized controlled trials.

### Limitations

While these results are promising, they should be interpreted with caution, as the sample size was small (69 participants, with 15 and 19 individuals with above-threshold depressive and anxiety symptoms, respectively), meaning that the performance metrics could be unreliable for imbalanced datasets. Future studies should include much larger samples (in Brazil and globally), in order to ensure, by extension, a higher positive class.

Another limitation could be a lack of sensitivity in the self-report instruments chosen as ground truth. While the PHQ-8 and GAD-7 have been validated in older cohorts [26,28], they were not specifically developed for an older population. Another depression screening tool, the Geriatric Depression Scale [36], was specifically designed for older adults; however, its specificity is suboptimal [37]. Moreover, it fails to account for common somatic symptoms such as the loss of appetite and sleep disturbances [38,39]. Another aspect relates to cultural differences in these self-report instruments. For example, research suggests that using a cut-off of ≥10 on the PHQ-8 might be too high for older individuals in Sweden [40]. Additionally, the Geriatric Depression Scale was found to not comprehensively capture depressive symptoms in older adults in the Indian context [41]. One possibility for future AI work in this space would be to include several self-report and screening measures for triangulation or to work with trained clinicians for an additional specialist benchmark.

Furthermore, it is important to note that there may be nuances in behavior, related to how the n-back performance changes during the session itself, that can also be used on top of metrics that aggregate the n-back performance across the entire duration of 1 session. While our results are based on the cohort level, future research could focus on the models’ ability to detect intraindividual changes related to shifts in symptom severity across time.

### Conclusions

In conclusion, this study successfully demonstrates the preliminary transferability of two AI models, one for depression and the other for anxiety classification, to a novel demographic of older Brazilian adults. The depression model showed acceptable performance, significantly outperforming a benchmark model based solely on age and gender, indicating its effective learning from n-back task–related biomarkers. This suggests its potential applicability across different cultures, particularly given the universality and nonverbal nature of the n-back task. The anxiety model, while showing promise in cross-sectional settings, needs further refinement for longitudinal effectiveness. This research contributes to the potential role of AI in addressing mental health issues, especially in populations like older Brazilians where depression is notably underdiagnosed. By integrating more culturally invariant biomarkers such as speech and fine-tuning these models with a diverse dataset, AI tools may become powerful aids in remote screening and monitoring, ultimately potentially contributing to a significant reduction in mental health underdiagnoses globally.

## Abbreviations

AI: artificial intelligence
AUC: area under the receiver operating characteristic curve
GAD: generalized anxiety disorder
GAD-7: Generalized Anxiety Disorder Assessment (7 items)
MDD: major depressive disorder
PHQ-8: Patient Health Questionnaire (8 items)
RADAR-MDD: Remote Assessment of Disease and Relapse in Major Depressive Disorder
RMT: remote measurement technology
